# Isoliquiritigenin Activates Nuclear Factor Erythroid-2 Related Factor 2 to Suppress the NOD-Like Receptor Protein 3 Inflammasome and Inhibits the NF-κB Pathway in Macrophages and in Acute Lung Injury

**DOI:** 10.3389/fimmu.2017.01518

**Published:** 2017-11-09

**Authors:** Qinmei Liu, Hongming Lv, Zhongmei Wen, Xinxin Ci, Liping Peng

**Affiliations:** ^1^Department of Respiration, The First Hospital of Jilin University, Changchun, China

**Keywords:** isoliquiritigenin, oxidative stress, inflammation, acute lung injury, AMP-activated protein kinase/nuclear factor erythroid-2 related factor 2, NOD-like receptor protein 3, NF-κB

## Abstract

Among the cellular response mechanisms, the nuclear factor erythroid-2 related factor 2 (Nrf2) pathway is considered a survival pathway that alleviates oxidative injury, while both the NOD-like receptor protein 3 (NLRP3) and NF-κB pathways are pro-inflammatory pathways that cause damage to cells. These pathways are implicated in the development and resolution of acute lung injury (ALI). Isoliquiritigenin (ISL), a flavonoid from the liquorice compound, is suggested to be a regulator of the above pathways, but the mechanisms of how the NLRP3/NF-κB pathway interacts with Nrf2 and its protective effects in ALI remain unknown. In the present study, ISL inhibited reactive oxygen species (ROS) generation and cytotoxicity induced by t-BHP and pro-inflammatory enzymes production induced by LPS in RAW 264.7 cells. Such cytoprotective effects coincided with the induction of AMP-activated protein kinase (AMPK)/Nrf2/antioxidant response element (ARE) signaling and the suppression of the NLRP3 and NF-κB pathways. Consistent with these findings, ISL treatment significantly alleviated lung injury in LPS-induced ALI mice, which was reflected by reductions in histopathological changes, pulmonary edema, and protein leakage. At the same time, the increased levels of inflammatory cell exudation and pro-inflammatory mediators, the enhanced production of ROS, myeloperoxidase, and malondialdehyde, and the depleted expression of GSH and superoxide dismutase induced by LPS were ameliorated by ISL. Furthermore, ISL notably activated AMPK/Nrf2/ARE signaling and inhibited LPS-induced NLRP3 and NF-κB activation in the lung. Moreover, although inhibition of the LPS-induced histopathological changes and ROS production were attenuated in Nrf2-deficient mice, the repression of the NLRP3 and NF-κB pathways by ISL was Nrf2-dependent and Nrf2-independent, respectively. In conclusion, our results are the first to highlight the beneficial role and relevant mechanisms of ISL in LPS-induced ALI and provide novel insight into its application.

## Introduction

In recent decades, redox homeostasis has been proven to play an essential role in the survival of aerobic organisms (1). The redox homeostasis is maintained by the balance between the generation of oxidants and free radicals, especially reactive oxygen species (ROS), and the scavenging capacity of endogenous antioxidant systems (2). However, after exposure to harmful stimuli, excessive production of ROS may overwhelm the antioxidant capacity, causing toxicity to cellular components including DNA, lipids, and proteins, which in turn exacerbates the oxidative burden and leads to oxidative stress (3). Increasing evidence indicates that oxidative stress is involved in the pathogenesis of diverse diseases, such as atherosclerosis, diabetes (4), carcinogenesis (5), and airway disorders (6). Furthermore, oxidative stress and inflammation, which are classical mechanisms of various diseases, are intimately correlated and orchestrated in a cycle that drives the central pathophysiological processes (7). Thus, the search for potential antioxidative pathways that can be regulated by endogenous or exogenous compounds may become prospective therapeutic targets for human diseases.

Among these antioxidative pathways, the pathway of the transcription factor nuclear factor erythroid-2 related factor 2 (Nrf2) is essential. Under basal conditions, Nrf2 is constitutively kept in the cytoplasm and degraded *via* binding to its main antagonist, Kelch-like ECH-associated protein 1 (Keap1). Upon oxidative stress, Nrf2 dissociates from Keap1 through diverse mechanisms, including the classical oxidative modification of cysteine thiols in Keap1 and the phosphorylation at specific amino acid residues of Nrf2 through several protein kinase pathways (8, 9). The intracellular energy sensor AMP-activated protein kinase (AMPK) is a kinase that works upstream of Nrf2 and that has attracted attention for its relationship with redox homeostasis and energy metabolism (10). Furthermore, a more detailed mechanism of AMPK-mediated Nrf2 activation may include the kinases Akt and glycogen synthase kinase 3 beta (GSK3β) (11). Nevertheless, no matter how Nrf2 dissociation occurs, after being released from Keap1, Nrf2 translocates into the nucleus and binds to antioxidant response elements (AREs) in the promoter region of a multiple cytoprotective genes (e.g., HO-1, GCLC, GCLM, NQO1). The induction of antioxidant cascades rescues the organisms from oxidative injury and meanwhile exerts a protective effect against inflammation (12, 13).

The interdependent relationship between oxidative stress and inflammation is supported by numerous studies (14, 15). Not only does inflammation induce oxidative stress but oxidative stress also accelerates inflammation through the activation of pro-inflammatory pathways, including the well-known NOD-like receptor protein 3 (NLRP3) inflammasome and nuclear factor kappa B (NF-κB) pathways (16, 17). NLRP3 inflammasome, the most fully characterized inflammasome, is an oligomeric molecular complex that can be activated by diverse “danger signals” (e.g., ATP and ROS) (18). Several important pro-inflammatory cytokines such as IL-1β and IL-18 are controlled by NLRP3 activation through recruitment of the adaptor protein ASC, activation of caspase-1 and cytokine precursors processing to mature forms (19, 20). Intriguingly, the NF-κB pathway does not merely act as a classical pro-inflammatory pathway with a conventional mode of action but also provides novel insight due to its critical role in the initial step of NLRP3 activation (21). Furthermore, numerous compounds have simultaneous anti-inflammatory and antioxidative effects, making the interaction between Nrf2 and NLRP3/NF-κB interesting (12, 13).

**Figure 1:**
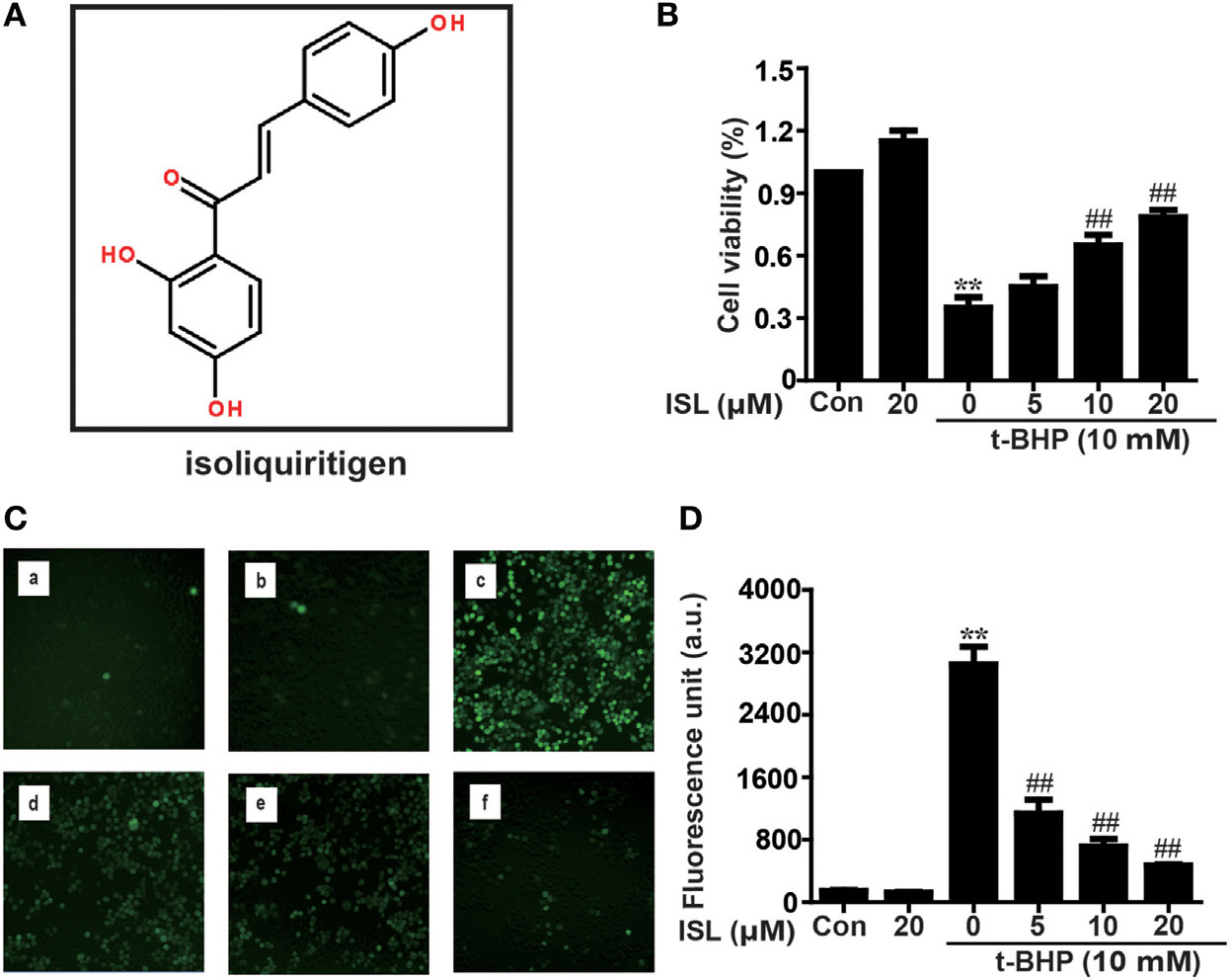
Effects of isoliquiritigenin (ISL) on t-BHP-induced oxidative injury in RAW 264.7 cells. **(A)** The chemical structure of ISL. **(B)** RAW 264.7 cells were treated with isoliquiritigenin (5, 10, 20 µM) for 18 h, and then subjected to t-BHP (10 mM) for 3 h. The cell viability was determined by a MTT assay. **(C,D)** RAW 264.7 cells were treated with isoliquiritigenin (5, 10, 20 µM) for 18 h and then stained with DCFH-DA (5 µM) for 40 min, followed by exposed to t-BHP (10 mM) for 5 min to induce reactive oxygen species (ROS). The ROS production was detected by microscope (original magnification 100×) and fluorescence microplate reader: (a) control, (b) isoliquiritigenin (20 µM), (c) t-BHP (10 mM), (d) t-BHP + isoliquiritigenin (5 µM), (e) t-BHP + isoliquiritigenin (10 µM), and (f) t-BHP + isoliquiritigenin (20 µM). Data were expressed as mean ± SEM. *n* = 5. *P < 0.05 and **P < 0.01 vs control group. ^#^*P* < 0.05 and ^##^*P* < 0.01 vs t-BHP group.

The pathogenesis of acute lung injury (ALI)/acute respiratory distress syndrome (ARDS), a major clinical syndrome with limited therapy and high mortality, is closely related to oxidative stress and inflammation (22, 23). Indeed, previous studies have investigated the protective effects of Nrf2 and the harmful effects of NLRP3 and NF-κB in the context of ALI/ARDS (24, 25). Isoliquiritigenin (ISL: Figure 1A), a flavonoid originating from *G. uralensis*, is an effective Nrf2 activator and NF-κB inhibitor, and recent evidence has shown that it can inhibit the activation of NLRP3 (26, 27). However, there is still no evidence to explain the mechanism of how the NLRP3/NF-κB pathway interacts with Nrf2 and whether that mechanism allows it to protect against ALI. Since infection by gram-negative bacteria containing lipopolysaccharide (LPS) is one of the most important causes of ALI/ARDS and leads to a substantial increase in oxidative stress and inflammation (28, 29), in the present study, we investigated the potential of ISL to protect against LPS-induced cell injury and ALI in mice and possible mechanisms underlying its effects.

## Materials and Methods

### Drug and Reagents

Isoliquiritigenin, purity > 98%, was obtained from Chengdu Pufei De Biotech Co., Ltd. Compound C (CC, a specific inhibitor of AMPK), brusatol (a specific inhibitor of Nrf2), and tert-butyl hydroperoxide (t-BHP) were obtained from Sigma-Aldrich (St. Louis, MO, USA). Dexamethasone was obtained from TianJin KingYork Group HuBei TianYao Pharmaceutical Co., Ltd. LPS (*Escherichia coli* 055:B5) and dimethylsulfoxide (DMSO) were obtained from Sigma Chemical Co. (St. Louis, MO, USA). Penicillin and streptomycin, fetal bovine serum (FBS), and Dulbecco’s modified Eagle’s medium (DMEM) for cell culture use were obtained from Invitrogen-Gibco (Grand Island, NY, USA). Myeloperoxidase (MPO), malondialdehyde (MDA), GSH, and superoxide dismutase (SOD) determination kit were provided by the Jiancheng Bioengineering Institute of Nanjing (Jiangsu, China). Mouse TNF-α, IL-1β, and IL-6 enzyme-linked immunosorbent assay (ELISA) kits were provided by Biolegend (San Diego, CA, USA). Antibodies against NLRP3, ASC, Caspase-1, IL-1β, p-AMPK, AMPK, p-GSK3β, GSK3β, p-IκBα, IκB, Nrf2, HO-1, NQO1, GCLM, GCLC, β-actin, and Lamin B were obtained from Cell Signaling (Boston, MA, USA) or Abcam (Cambridge, MA, USA). The horseradish peroxidase-conjugated anti-rabbit or anti-mouse IgG were obtained from Protein-Tech (Boston, MA, USA). Unless specifically stated, all other chemicals were obtained from Sigma-Aldrich (St. Louis, MO, USA).

### Cell Culture

RAW 264.7 cells were obtained from the China Cell Line Bank (Beijing, China). The cells were cultured in DMEM supplemented with 10% FBS, 100 U/ml of penicillin and 100 U/ml of streptomycin in a 5% CO_2_-humidified atmosphere at 37°C prior to experiments.

### Cell Viability

Cell viability were determined using an MTT (3-(4,5-dimethylthiazol-2-yl)-2,5-diphenyltetrazolium bromide) assay. RAW 264.7 cells were plated at a density of 2 × 10^4^ cells per well in a 96-well plate and incubated at 37°C for 18 h. The cells were treated with various concentrations of ISL (5, 10, 20 µM) for 18 h with or without brusatol (a specific inhibitor of Nrf2) and then exposed to t-BHP (10 mM) for 3 h. After that, 20 µL of MTT (0.5 mg/mL) was added into each well, and the cells were incubated for another 4 h. The formazan crystals were solubilized in 150 µL DMSO, and the absorbance was measured at 570 nm on a microplate reader. The relative cell viability was calculated as a percentage against the untreated group.

### ARE Promoter Activity

RAW 264.7 cells were plated at a density of 1.5 × 10^4^ cells per well in a 96-well plate and cultured to reach 75% confluence. Using the Viafect transfection reagent (Invitrogen, Carlsbad, CA, USA), pGL4.37 (luc2P/ARE/Hygro vector) and pGL4.74 (hRluc/TK vector) plasmids were transfected into cells. After treatment of ISL at different concentrations (5, 10, 20 µM) for 18 h or at 20 µM for 1, 3, 6, and 18 h, a dual-luciferase reporter assay system (Dual-Glo^®^ Luciferase Assay System) was put into use to determine the ARE-driven promoter activity.

### Animal

Wild-type (WT) C57BL/6 mice (18–20 g) were purchased from Liaoning Changsheng Technology Industrial Co., Ltd. (Certificate SCXK2010-0001; Liaoning, China), and Nrf2^−/−^ (knockout) C57BL/6 mice were purchased from The Jackson Laboratory (Bar Harbor, ME, USA). The animals were housed in certified, standard laboratory cages and fed with food and water *ad libitum* before using for experiments. All of the experiments were approved by Animal Use Committee of Jilin University (license number: SYXK (Ji) 2014-0006), in accordance with International Guiding Principles for Biomedical Research Involving Animals.

### Grouping and Establishment of LPS-Induced ALI Mouse Model

To establish ALI model, an intranasal instillation of LPS (0.5 mg/kg) was administered to the mice. In the treated groups, mice received a single dose of ISL or dexamethasone (Dex) *via* intraperitoneal (i.p.) route 1 h before the administration of LPS. Briefly, WT were divided into five groups randomly: (1) control, (2) ISL (30 mg/kg), (3) LPS (0.5 mg/kg), (4) LPS + ISL (30 mg/kg), and (5) LPS + dexamethasone (5 mg/kg). For further comparison between WT and Nrf2^−/−^ (knockout) mice, they were, respectively, divided into four groups: (1) control, (2) ISL (30 mg/kg), (3) LPS (0.5 mg/kg), and (4) LPS + ISL (30 mg/kg). At 12 h after LPS treatment, the mice were sacrificed by diethyl ether.

### Isolation of Peritoneal Macrophages

Mice received starch broth of 6% *via* intraperitoneal (i.p.) route 2 or 3 days before isolation. Serum-free DMEM was injected into the abdominal cavity of the mice, while the abdomen was kneaded softly for 2 min. The culture medium was drawn out and collected, and then centrifuged for 5 min at 1,500 rpm. Sediment cells were resuspended in DMEM, which regulated the concentration of the cells at 2 × 10^6^ cells/ml. The cells were cultivated in 96-well plates at 37°C for 2 h until they had adhered, then the cultivation holes were washed with PBS. The adherent cells were peritoneal macrophages.

### Evaluation of Histological Changes

For the histological analysis of lung tissue, mice were euthanized and the lower lobes from left lungs were fixed in 4% formalin, followed by dehydrated with ethanol. Subsequently, 5 µm sections were cut after paraffin embedding, and stained with hematoxylin and eosin (H&E) according to previous description (30). The H&E-stained sections were observed under a light microscope to evaluate pathological changes. At the same time, we applied a standard assessment method to judge the lung injury (31). In detail, lung injury was scored based on edema, neutrophil infiltration, hemorrhage, bronchiole epithelial desquamation, and hyaline membrane formation, and five visual fields were observed for each slice. The works were performed in a blinded manner. A score scaled from 0 to 4 represents the severity: 0 for no damage, 1 for mild damage, 2 for moderate damage, 3 for severe damage, and 4 for very severe damage.

### Measurement of Lung Wet/Dry Weight Ratios

Right lungs of the mice were separated by blunt dissection, and the wet weights were measured. And then the lungs were dried in an oven continuously at 60°C for 3 days to determine the dry weights. Ultimately, we calculated the ratios of wet/dry weight to evaluate the degree of pulmonary edema.

### Bronchoalveolar Lavage Fluid (BALF) Analysis

Trachea was exposed after the mice were euthanized. Gently we injected 0.5 mL PBS into the trachea and aspirate the liquid for three times to obtain BALF (32). BALF were centrifuged to pellet cells, the supernatants were used to measure total protein concentration by BCA (Bicinchoninic acid) method. The cells were then lysed by ACK Lysis Buffer for 5 min, washed twice with ice-cold PBS, and then centrifuged again at 3,000 rpm for 10 min at 4°C. After that, sediment cells were resuspended in PBS to count total cell number with a hemocytometer or conduct Wright–Giemsa staining using a cytospin and count differential inflammatory cell numbers, as well as measure the ROS level. Meanwhile, the supernatants were conserved at −80°C to measure cytokine levels by ELISA.

### Cytospins and Wright–Giemsa Staining

Cells from BALF were spun onto glass slides by a cytospin. After the slide was mixed in methyl alcohol, Wright–Giemsa A was added onto it for 1 min, and then Wright–Giemsa B was added on top of Wright–Giemsa A for 5 min. The dye liquor was washed off gently by running water, followed by drying and observation under a microscope. To count differential inflammatory cell number, 300 cells in total were counted, and 100 of the cells in each microscopic field were scored. The mean number of cells per field was reported.

### Enzyme-Linked Immunosorbent Assay

Applying a commercially available mouse ELISA kits, the levels of pro-inflammatory cytokines IL-1β, IL-6, and TNF-α in BALF were detected. The absorbance was read at 450 nm with a microplate reader.

### Measurements of MPO, MDA, GSH, and SOD Contents

Right lungs of the mice were excised and homogenized in saline. The levels of MPO, MDA, GSH, and SOD were determined by commercially available test kits according to the manufacturer’s kit protocols.

### Measurement of ROS Generation

Reactive oxygen species scavenging activity of ISL was assayed with the oxidant-sensitive fluorescent probe, DCFH-DA (20, 70-Dichlorofluorescein diacetate). ISL-treated cells with or without brusatol were stained with DCFH-DA (5 µM) for 40 min prior to t-BHP (10 mM) for 5 min, or stimulated with LPS (1 µg/mL) for 24 h prior to DCFH-DA (5 µM) for 40 min, the fluorescence intensity was read by a microplate reader at an excitation wavelength of 488 nm and an emission wavelength of 535 nm. As well, resuspended cells in BALF were stained with the same fluorescent dye, but the ROS levels were determined by both the microplate reader and flow cytometry.

### Isolation of Nuclear and Cytosolic Fractions

Cytoplasmic and nuclear extracts were prepared using an NE-PER Nuclear and Cytoplasmic Extraction Reagents kit (Pierce Biotechnology, Rockford, IL, USA), following the manufacturer’s instructions. All steps were performed on ice.

### Western Blots

For western blot analysis, homogenized lung tissues and treated cells were lysed in RIPA buffer with protease and phosphatase inhibitors for 30 min, followed by centrifugation at 12,000 rpm for 10 min at 4°C. The supernatants were collected, and BCA method was used to determine protein concentration. Protein samples were separated by 10–12.5% SDS-PAGE, and were transferred to a PVDF membrane. The membrane was blocked in 5% skim milk at room temperature for 1 h, blotted with each primary antibody (1:1,000) overnight at 4°C and the corresponding secondary antibody (1:5,000) at room temperature for 1 h. Finally the blots were visualized with an enhanced chemiluminescence western blot detection system (Amersham Pharmacia Biotech, Piscataway, NJ, USA) and band intensities were quantified using Image J gel analysis software.

### Statistical Analysis

The data were analyzed using SPSS19.0 (IBM) and expressed as mean ± SEM. The statistical analysis of comparison among groups was performed with the one-way analysis of variance, whereas multiple comparisons were made using the LSD method. Statistical significance was accepted when *P* < 0.05 or *P* < 0.01.

## Results

### ISL Inhibited Cell Death and ROS Production in t-BHP-Treated RAW 264.7 Cells *via* a Nrf2-Dependent Mechanism

First, the cytoprotective effects of ISL were confirmed in t-BHP-treated RAW 264.7 cells. Pretreatment with ISL (18 h) attenuated the cell death induced by t-BHP in an MTT assay (Figure 1B). Meanwhile, incubation with t-BHP significantly raised the intracellular ROS levels, which were inhibited by pretreatment with ISL (Figures 1C,D). These results suggest that ISL may alleviate cytotoxicity through its intracellular ROS scavenging activity. However, brusatol pretreatment abolished the protective effects of ISL on cell viability and ROS (Figures S1A,B in Supplementary Material), which indicated that the cytoprotective and antioxidant effects of ISL in t-BHP-treated RAW cells were dependent on Nrf2.

### ISL Upregulated AMPK/Nrf2/ARE Signaling and Related Antioxidant Enzyme Expression Levels in RAW 264.7 Cells

**Figure 2:**
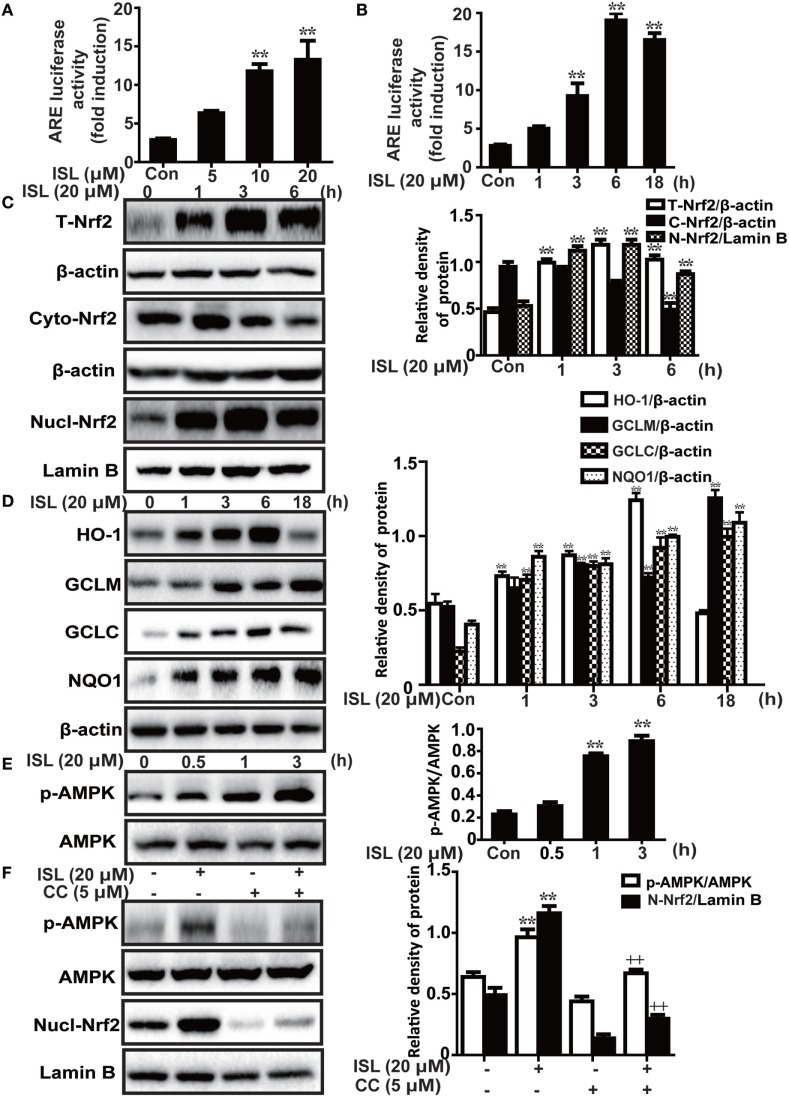
Effects of isoliquiritigenin (ISL) on AMP-activated protein kinase (AMPK)/nuclear factor erythroid-2 related factor 2 (Nrf2) pathway in RAW 264.7 cells. **(A,B)** The effect of isoliquiritigenin on antioxidant response element (ARE) luciferase activity for different concentrations (5, 10, 20 µM) or time points (1, 3, 6, or 18 h) in RAW 264.7 cells was determined by a dual-luciferase reporter assay system. **(C)** RAW 264.7 cells were treated with isoliquiritigenin (20 µM) for different time points (1, 3, 6 h). Total, nuclear and cytoplasmic levels of Nrf2 were determined by western blot. **(D)** RAW 264.7 cells were treated with isoliquiritigenin (20 µM) for different time points (1, 3, 6, or 18 h). The protein expressions of HO-1, GCLC, GCLM, and NQO1 were determined by western blot. **(E)** RAW 264.7 cells were treated with isoliquiritigenin (20 µM) for different time points (0.5, 1, 3 h). The protein expressions of p-AMPK and AMPK were determined by western blot. **(F)** RAW 264.7 cells were treated with CC (compound C, an inhibitor of AMPK, 5 µM) for 24 h, and then subjected to isoliquiritigenin for 3 h. The protein expressions of p-AMPK, AMPK, and nuclear levels of Nrf2 were determined by western blot. The results of western blot were expressed as densitometry quantitation using β-actin as an internal control for total or cytoplasmic proteins and Lamin B for nuclear proteins. Data were expressed as mean ± SEM. *n* = 5. *P < 0.05 and **P < 0.01 vs control group, ^+^*P* < 0.05 and ^++^*P* < 0.01 vs ISL only group.

Under conditions of oxidative stress, the expressions of antioxidant enzymes regulated by Nrf2, such as HO-1, GCLM, GCLC, and NQO1, are clearly increased. As expected, we discovered that ISL significantly increased the ARE-mediated transcriptional activities of antioxidant enzyme genes (Figures 2A,B). In addition, the results of western blot showed that ISL increased the protein expression of total Nrf2 and HO-1, GCLM, GCLC, and NQO1 in a time-dependent manner, as well as resulted in an increase in the nuclear expression and a decrease in cytoplasmic expression of Nrf2 (Figures 2C,D).

Furthermore, previous studies have shown that the AMPK pathway may work upstream of Nrf2. In the current study, we also found that ISL increased the phosphorylation of AMPK slightly prior to the induction of Nrf2 (Figure 2E), while pretreatment with an AMPK inhibitor, CC decreased the ISL-induced Nrf2 activation (Figure 2F). The results suggest that in ISL-mediated Nrf2 activation, AMPK signaling works as an upstream regulator.

### ISL Protected RAW 264.7 Cells from LPS-Induced Inflammation *via* both Nrf2-Dependent and Nrf2-Independent Mechanisms

**Figure 3:**
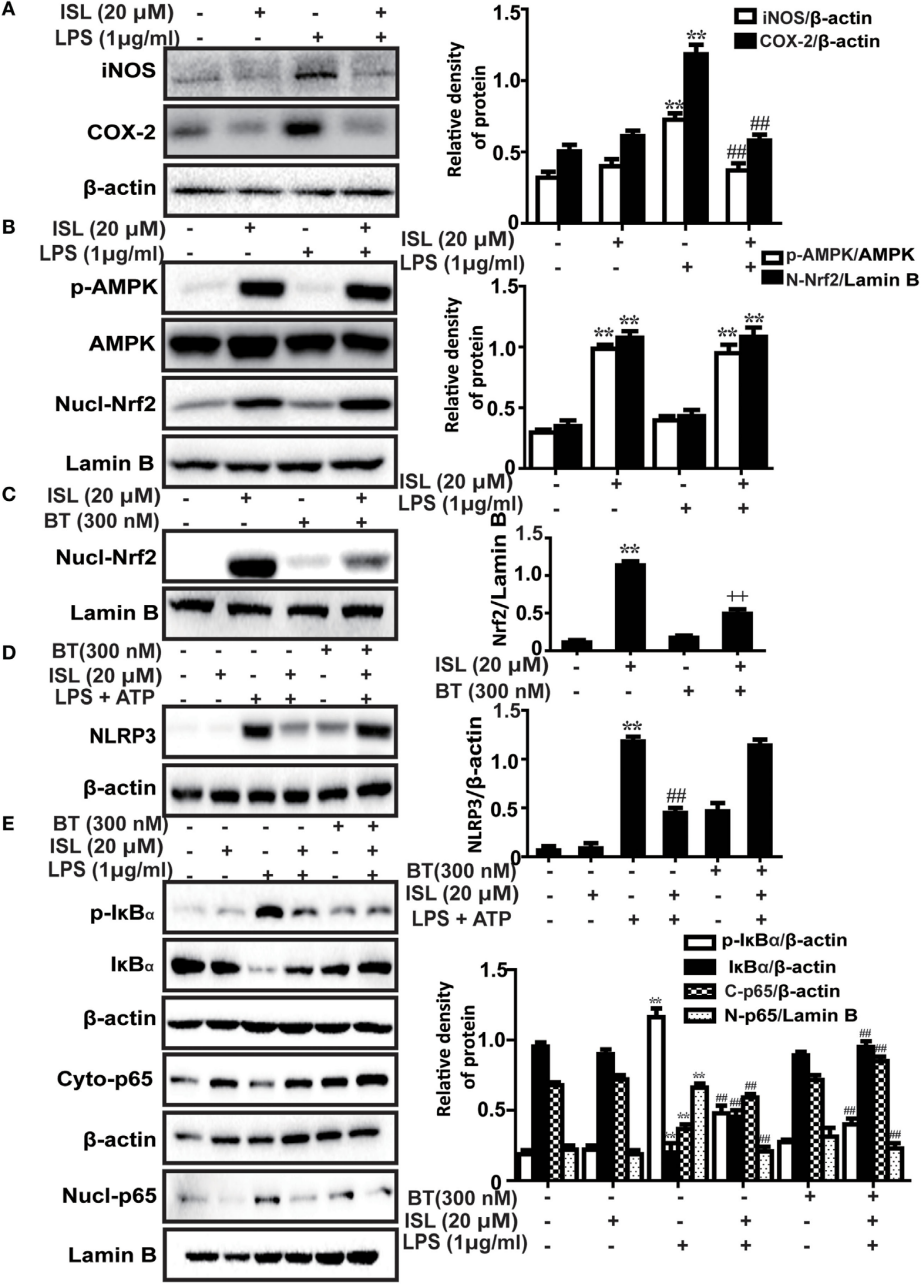
Effects of isoliquiritigenin (ISL) on lipopolysaccharide (LPS)-stimulated inflammatory responses in RAW 264.7 cells. **(A)** RAW 264.7 cells were treated with isoliquiritigenin (20 µM) for 1 h and then exposed to LPS (1 µg/mL) for 24 h. The protein expressions of inducible nitric oxide synthase and cyclooxygenase-2 were determined by western blot. **(B)** RAW 264.7 cells were treated with isoliquiritigenin (20 µM) for 1 h and then exposed to LPS (1 µg/mL) for 6 h. The protein expressions of p-AMPK, AMP-activated protein kinase (AMPK), and nuclear levels of nuclear factor erythroid-2 related factor 2 (Nrf2) were determined by western blot. **(C)** RAW 264.7 cells were treated with BT (brusatol, an inhibitor of Nrf2, 300 nM) for 1 h and then subjected to isoliquiritigenin (20 µM) for 6 h. The inhibition of Nrf2 expression was determined by western blot. **(D)** RAW 264.7 cells were treated with BT (300 nM) for 1 h, and then subjected to isoliquiritigenin (20 µM) for 1 h prior to incubation with LPS (1 µg/mL) for another 6 h followed by ATP (5 mM) stimulation for 45 min. The protein expression of NOD-like receptor protein 3 (NLRP3) were determined by western blot. **(E)** RAW 264.7 cells were treated with BT (300 nM) for 1 h, and then subjected to isoliquiritigenin (20 µM) for 6 h prior to incubation with LPS (1 µg/mL) for another 1 h. The protein expressions of p-IκBα and IκBα, nuclear and cytoplasmic levels of p65 were determined by western blot. The results were expressed as densitometry quantitation using β-actin as an internal control for total or cytoplasmic proteins and Lamin B for nuclear proteins. Data were expressed as mean ± SEM. *n* = 5. *P < 0.05 and **P < 0.01 vs control group. ^#^*P* < 0.05 and ^##^*P* < 0.01 vs LPS group, ^+^*P* < 0.05 and ^++^*P* < 0.01 vs ISL only group.

Recently, a series of studies have shown that the potent anti-inflammatory effect of ISL is partly derived from the inhibition of the caspase-1/IL-1β and NF-κB pathways. However, due to the crosstalk between Nrf2 and anti-inflammatory pathways, we deemed it worthwhile to investigate whether the Nrf2 activator ISL exerts anti-inflammatory effects through Nrf2 signaling. We confirmed that ISL also inhibited the induction of two important pro-inflammatory enzymes inducible nitric oxide synthase (iNOS) and cyclooxygenase-2 (COX-2) in RAW 264.7 cells (Figure 3A). The results showed that AMPK/Nrf2 signaling was activated in the ISL-treated group after stimulation with LPS, preliminarily suggesting that ISL does exert its anti-inflammatory effects through Nrf2 signaling (Figure 3B). Subsequently, we observed that the protein expression levels of NLRP3, p-IκBα and nuclear NF-κB (p65) were significantly increased by the combination of LPS and ATP or by just LPS stimulation, while this increase was blocked by pretreatment with ISL. Furthermore, we used a specific inhibitor of Nrf2, brusatol, to inhibit ISL-induced Nrf2 activation (Figure 3C), and the inhibitory effects of brusatol on the antioxidant enzymes regulated by Nrf2 were also investigated (Figure S2 in Supplementary Material). As might be expected, ISL failed to inhibit the increase in NLRP3 expression after brusatol pretreatment (Figure 3D). Nevertheless, we were surprised to discover that the inhibitory effects on the NF-κB pathway were not affected (Figure 3E). These results suggest that ISL suppresses the LPS-induced inflammation in RAW 264.7 cells through the inhibition of NLRP3 in a Nrf2-dependent way and the suppression of the NF-κB pathway in a Nrf2-independent way.

### ISL Reduced the Severity of LPS-Induced ALI

**Figure 4:**
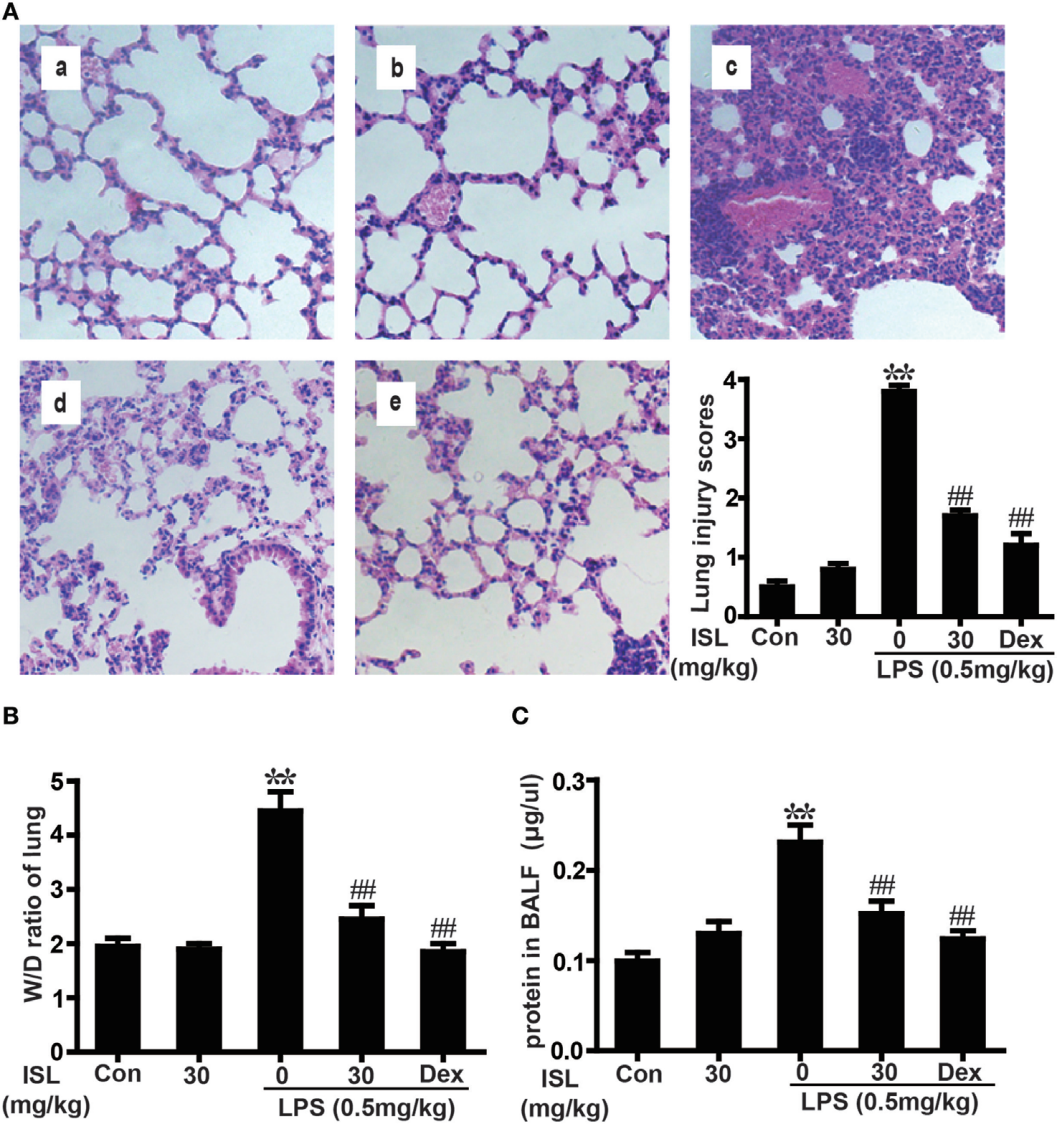
Effects of isoliquiritigenin (ISL) on lung injury in lipopolysaccharide (LPS)-induced ALI mice. **(A)** Lung tissues from each experimental group (*n* = 5) were obtained at 12 h after LPS administration, prepared as tissue sections and subjected to H&E staining (original magnification 200 × ): (a) control, (b) isoliquiritigenin (30 mg/kg), (c) LPS (0.5 mg/kg), (d) LPS + ISL (30 mg/kg), and (e) LPS + Dex (5 mg/kg). As well, lung mean injury score was determined according to a five-point scale that was previously described. **(B)** Lung wet-to-dry weight ratio was determined by the method mentioned above. **(C)** Total protein concentration in bronchoalveolar lavage fluid (BALF) was measured by a BCA method. Data was expressed as mean ± SEM. *n* = 5. *P < 0.05 and **P < 0.01 vs control group. ^#^*P* < 0.05 and ^##^*P* < 0.01 vs LPS group.

Previous studies have shown that the pathological characteristics of LPS-induced ALI in a mouse model are fairly similar to those in human ALI (33). Therefore, LPS instillation was applied to induce ALI in our study and to investigate the protective effects of ISL. To observe the tissue damage, the lower lobes of the left lungs were taken for histology 12 h after the LPS challenge. The administration of LPS led to diffuse pathological changes in the lung, which were characterized by alveolar congestion and inflammatory cell infiltration. However, pretreatment with ISL significantly reduced these changes, just as the positive control drug dexamethasone did. The evaluation of lung injury severity was also performed by calculating a lung injury score (Figure 4A). In accordance with the pathological analysis, the lung wet/dry weight ratio and total protein concentration in BALF showed similar results (Figures 4B,C). These results indicate that ISL effectively alleviates LPS-induced pathological changes in ALI.

### ISL Alleviated LPS-Induced Oxidative Stress in ALI

**Figure 5:**
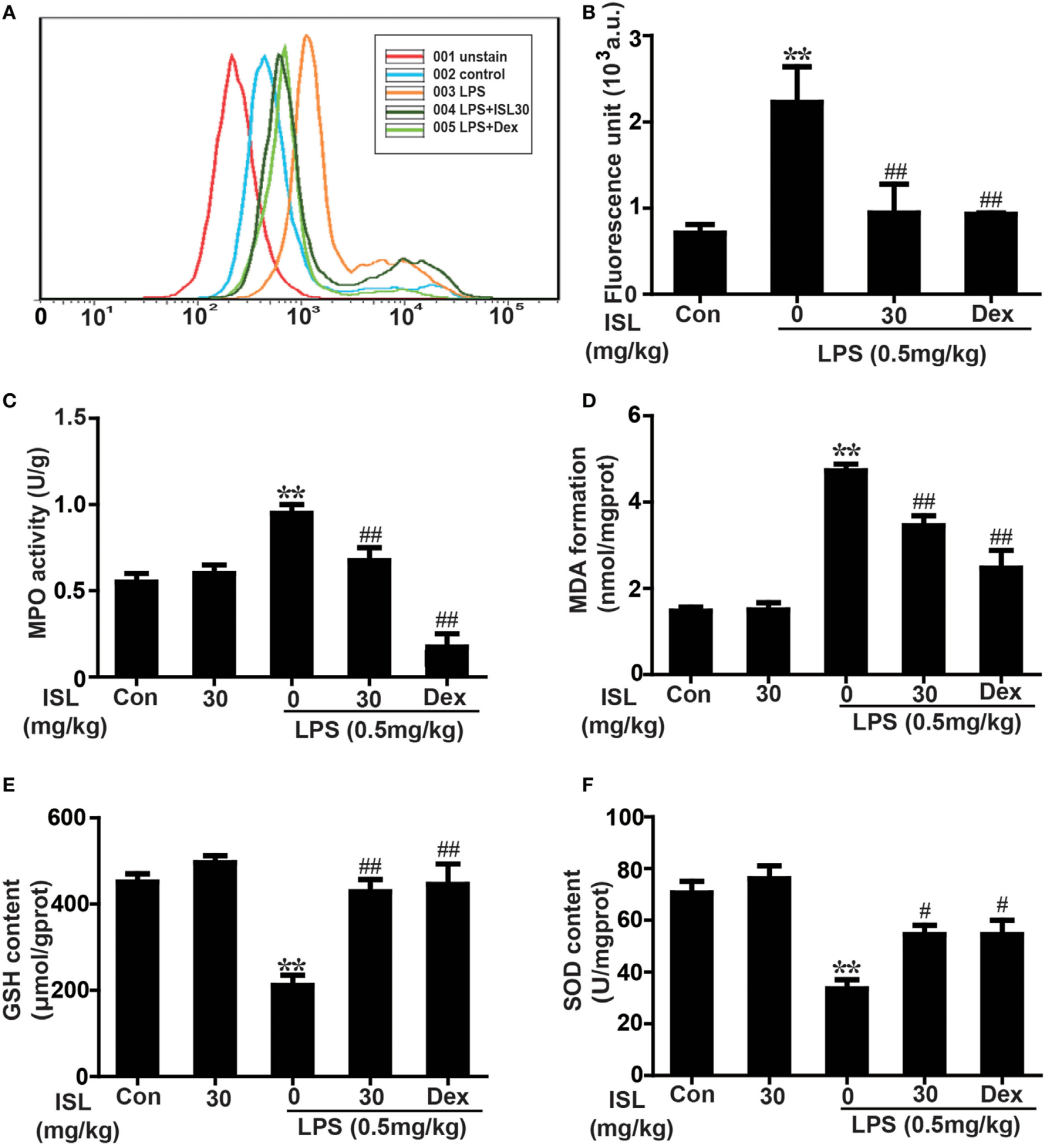
Effects of isoliquiritigenin on oxidative stress in lipopolysaccharide (LPS)-induced ALI mice. **(A)** Reactive oxygen species (ROS) production in bronchoalveolar lavage fluid (BALF) was analyzed with DCFH-DA by flow cytometry. **(B)** ROS generation in BALF was detected with DCFH-DA by fluorescence microplate reader and expressed as fluorescence unit. **(C–F)** Myeloperoxidase (MPO) activation, malondialdehyde (MDA) formation, GSH, and superoxide dismutase (SOD) levels in lung tissues was measured by commercially available test kits according to manufacturer’s kit protocols. Data was expressed as mean ± SEM. *n* = 5. ^*^P < 0.05 and **P < 0.01 vs the control group. ^#^*P* < 0.05 and ^##^*P* < 0.01 vs the LPS group.

To further confirm whether ISL combats LPS-induced ALI through its antioxidative effect, we examined the levels of ROS in different groups. After 12 h, stimulation with LPS significantly increased the ROS levels of sediment cells in BALF, and ISL significantly reduced this increase (Figures 5A,B). Additionally, we measured indexes of oxidative stress, including MPO, a marker of neutrophil accumulation that can cause tissue injury by itself or through derived oxidants; MDA, a product of lipid peroxidation considered as a marker of oxidative stress; and glutathione (GSH) and SOD, two essential antioxidant factors (34–37). The results showed that pretreatment with ISL markedly decreased LPS-induced MPO production and MDA formation and reduced the depletion of GSH and SOD (Figures 5C–F). These results reveal that ISL attenuates lung injury through the inhibition of oxidative stress.

### ISL Repressed the LPS-Induced Recruitment of Inflammatory Cells and Production of Pro-inflammatory Mediators

**Figure 6:**
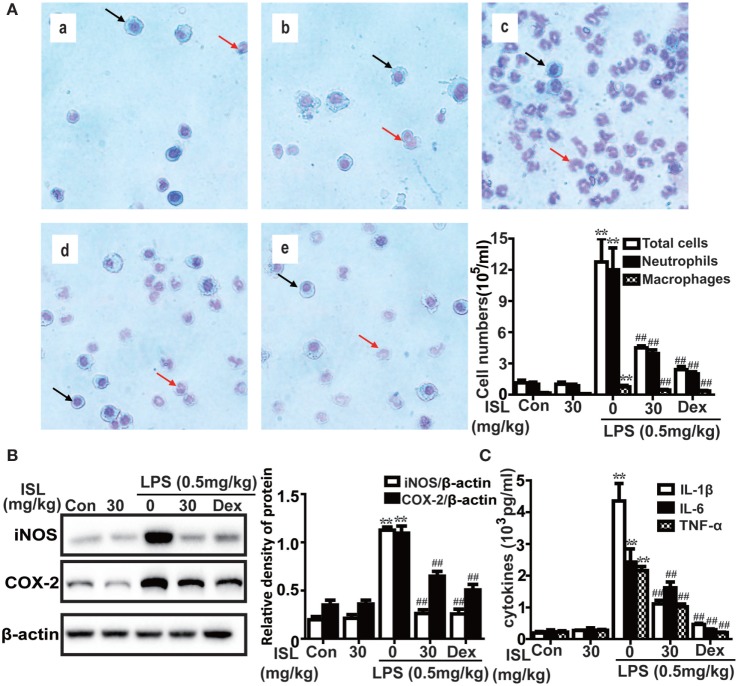
Effects of isoliquiritigenin (ISL) on inflammation in lipopolysaccharide (LPS)-induced ALI mice. **(A)** Wright–Giemsa staining was used to display morphology of different inflammatory cells (magnification ×400, black arrows: macrophages; red arrows: neutrophils): (a) control, (b) isoliquiritigenin (30 mg/kg), (c) LPS (0.5 mg/kg), (d) LPS + ISL (30 mg/kg), and (e) LPS + Dex (5 mg/kg). As well, the numbers of total cells, neutrophils, and macrophages were counted with a hemocytometer to determine the inflammatory cell infiltration. **(B)** Lung tissues from different experimental groups were obtained at 12 h after LPS administration. Total protein was extracted from lung homogenization. The expressions of inducible nitric oxide synthase (iNOS) and cyclooxygenase-2 (COX-2) were determined by western blot and expressed as densitometry quantitation using β-actin as an internal control. **(C)** The levels of proinflammatory cytokines (TNF-α, IL-6, and IL-1β) in bronchoalveolar lavage fluid were detected by enzyme-linked immunosorbent assay. Data was expressed as mean ± SEM. *n* = 5. *P < 0.05 and **P < 0.01 vs control group. ^#^*P* < 0.05 and ^##^*P* < 0.01 vs LPS group.

The number of total and differential inflammatory cells as well as the levels of pro-inflammatory cytokines in BALF are involved in the pathogenesis of LPS-induced ALI and indicate the severity of lung inflammation. As evidenced by Wright–Giemsa staining and cell counting, LPS induced a significant increase in the number of total cells, neutrophils, and macrophages in BALF, while pretreatment with ISL caused a notable reduction in cell number compared with that after LPS treatment alone (Figure 6A). Moreover, the results showed that ISL inhibited the production of COX-2 and iNOS in the lung tissue, and TNF-α, IL-1β, and IL-6 in BALF (Figures 6B,C). These results suggest that ISL alleviates lung injury by inhibiting the recruitment of inflammatory cells and the production of pro-inflammatory mediators in LPS-induced ALI.

### ISL Activated the AMPK/Nrf2 Signaling and Its Downstream Antioxidant Enzymes in LPS-Induced ALI

**Figure 7:**
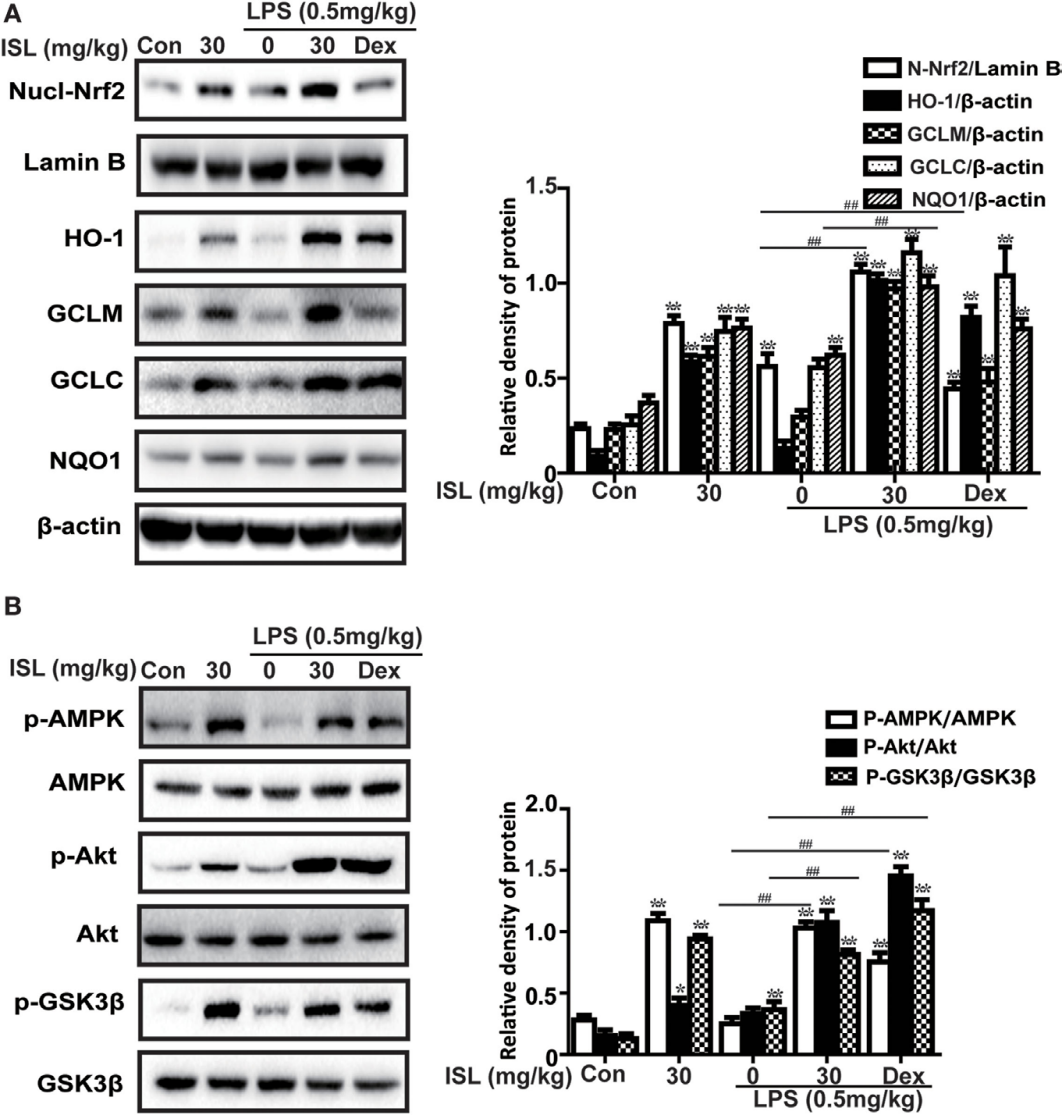
Effects of isoliquiritigenin on AMP-activated protein kinase (AMPK)/nuclear factor erythroid-2 related factor 2 (Nrf2) pathway in lipopolysaccharide (LPS)-induced ALI mice. Lung tissues from different experimental groups were obtained at 12 h after LPS administration. Total protein, cytoplasmic and nuclear extracts were extracted from lung homogenization. The expressions of different protein were determined by western blot and expressed as densitometry quantitation using β-actin as an internal control for total or cytoplasmic proteins and Lamin B for nuclear proteins. **(A)** Nuclear levels of Nrf2 and expressions of Nrf2-target genes (HO-1, GCLM, GCLC, NQO1). **(B)** The expressions of p-AMPK, AMPK, p-GSK3β, GSK3β, and p-Akt. Data were expressed as mean ± SEM. *n* = 5. *P < 0.05 and **P < 0.01 vs the control group. ^#^*P* < 0.05 and ^##^*P* < 0.01 vs the LPS group.

Various cellular and mouse models have been used to investigate the protective effects of Nrf2 against ALI/ARDS (38, 39). As expected, ISL treatment also evoked a notable increase in the expression of nuclear Nrf2 and its target enzymes (e.g., HO-1, GCLM, GCLC, and NQO1) in lung homogenates (Figure 7A). Moreover, the results showed that ISL markedly increased the phosphorylation of AMPK, GSK3β, and Akt (Figure 7B), which have been confirmed to be upstream regulators of Nrf2 (11). Such findings suggest that ISL may protect against LPS-induced ALI through the upregulation of the AMPK/Nrf2 signaling.

### ISL Suppressed the NLRP3 Inflammasome and NF-κB Pathways in LPS-Induced ALI

**Figure 8:**
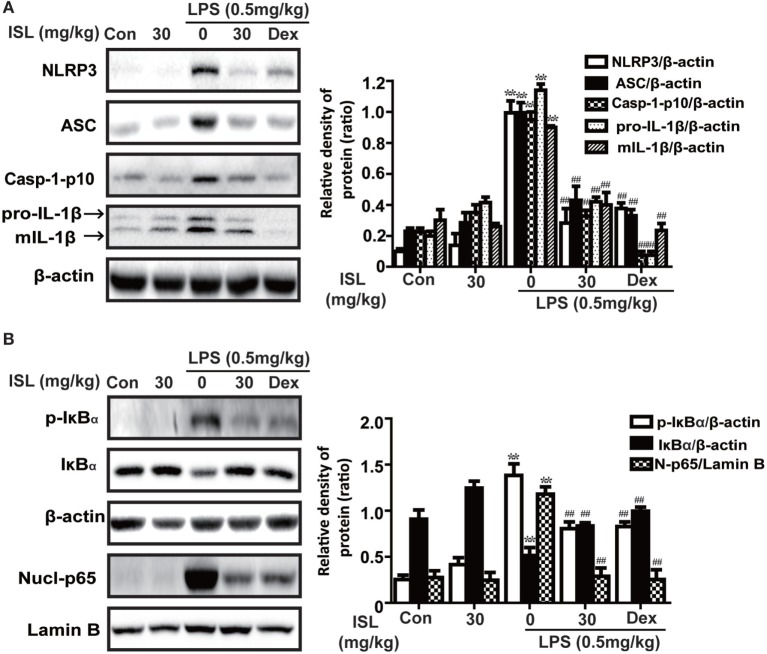
Effects of isoliquiritigenin on NOD-like receptor protein 3 (NLRP3) inflammasome and NF-κB pathways in lipopolysaccharide (LPS)-induced ALI mice. Lung tissues from different experimental groups were obtained at 12 h after LPS administration. Total protein, cytoplasmic, and nuclear extracts were extracted from lung homogenization. The expressions of different protein were determined by western blot and expressed as densitometry quantitation using β-actin as an internal control for total or cytoplasmic proteins and Lamin B for nuclear proteins. **(A)** The expressions of NLRP3, ASC, Caspase-1-p10, and pro- and mature IL-1β. **(B)** The protein expressions of p-IκBα and IκBα, and nuclear levels of p65. Data was expressed as mean ± SEM. *n* = 5. *P < 0.05 and **P < 0.01 vs the control group. ^#^*P* < 0.05 and ^##^*P* < 0.01 vs the LPS group.

Accumulated evidence has revealed that the NLRP3 inflammasome and NF-κB pathways are essential for the full development of ALI. Hence, we analyzed the expression levels of proteins in the NLRP3 and NF-κB pathways. The results showed that in the lung tissue, the administration of LPS substantially increased the protein expression levels of NLRP3, ASC, and caspase-1, which have been proven to be three important components of the inflammasome. ISL successfully suppressed the activation of the NLRP3 inflammasome induced by LPS. In agreement with this, the increase in both pro-IL-1β (31 kDa) and mature IL-1β (17.5 kDa) after the LPS challenge was blocked by ISL pretreatment (Figure 8A). Moreover, ISL reduced the LPS-stimulated increase in p-IκBα and the nuclear expression of NF-κB, both of which are important proteins in the NF-κB pathway (Figure 8B).

### Nrf2 Dependency of ISL in Repressing Oxidative Stress and Inflammation in LPS-Induced ALI

**Figure 9:**
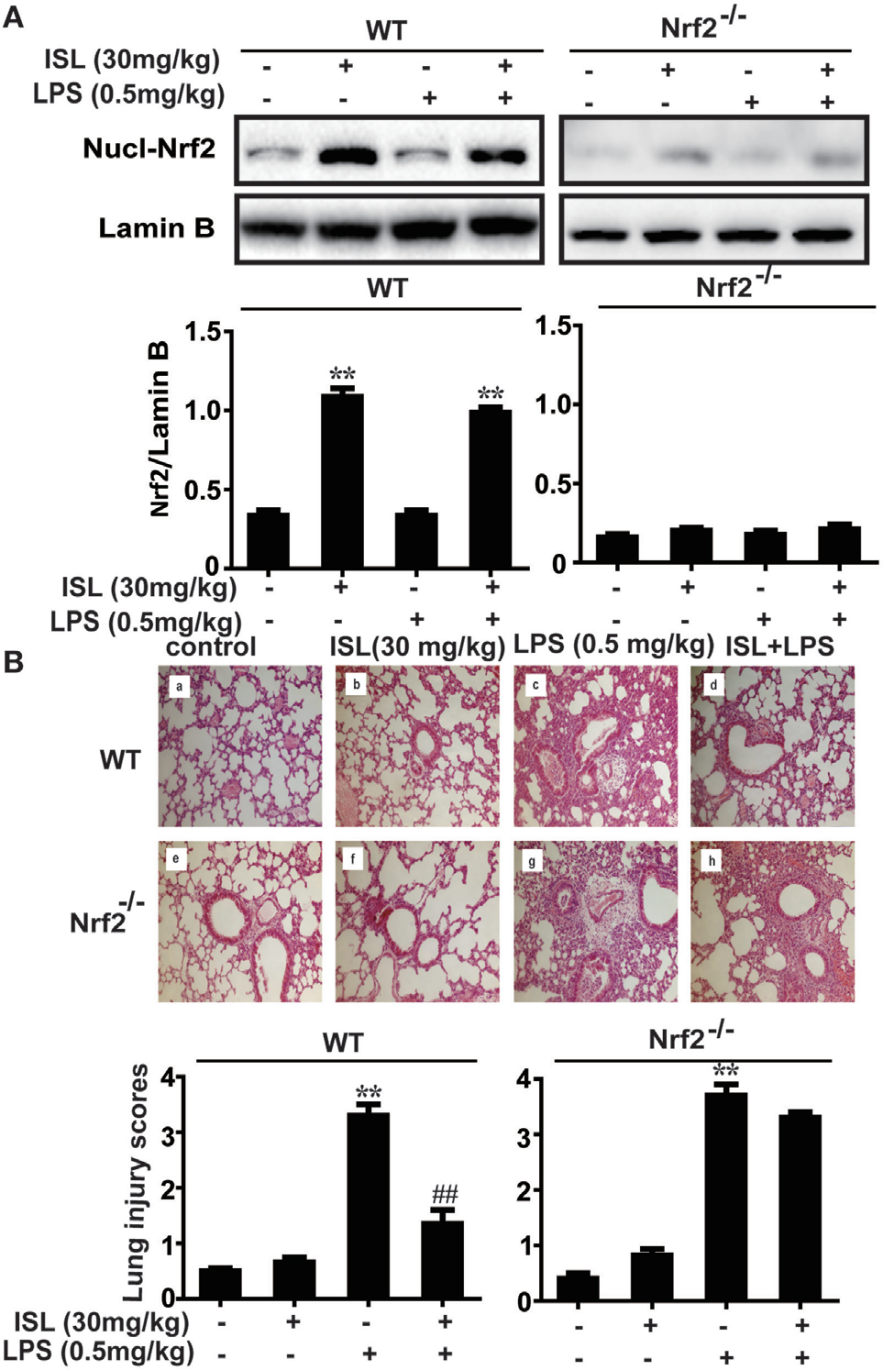
Nuclear factor erythroid-2 related factor 2 (Nrf2) dependence of protective effects mediated by isoliquiritigenin in lipopolysaccharide (LPS)-induced ALI mice. Wild-type (WT) and Nrf2^−/−^ mice were used to established the LPS-induced ALI model. **(A)** To confirm the knockout of Nrf2, nuclear levels of Nrf2 in lung homogenization were determined by western blot. The results of western blot were expressed as densitometry quantitation using Lamin B as an internal control. **(B)** Lung tissues from each experimental group (*n* = 5) were obtained at 12 h after LPS administration and processed for histological evaluation by H&E staining (original magnification ×200). As well, lung mean injury score was determined according to a five-point scale that was previously described.

**Figure 10:**
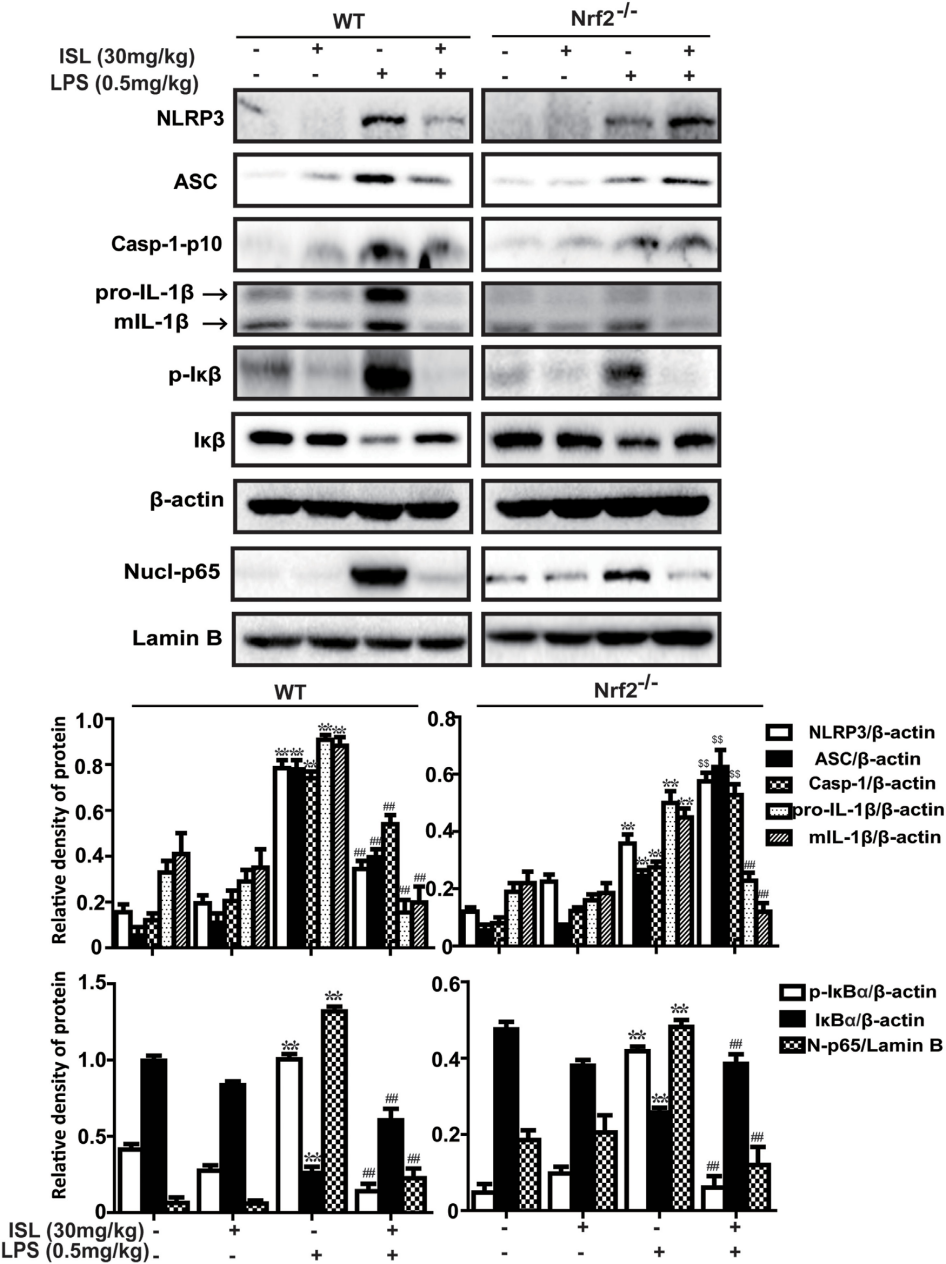
Nuclear factor erythroid-2 related factor 2 (Nrf2) dependence of NOD-like receptor protein 3 (NLRP3)/NF-κB inhibition mediated by isoliquiritigenin in lipopolysaccharide (LPS)-induced ALI mice. Wild-type (WT) and Nrf2^−/−^ mice were used to established the LPS-induced ALI model, and lung tissues from each experimental group (*n* = 5) were obtained at 12 h after LPS administration. Total protein, cytoplasmic, and nuclear extracts were extracted from lung homogenization. The expressions of different protein were determined by western blot and expressed as densitometry quantitation using β-actin as an internal control for total or cytoplasmic proteins and Lamin B for nuclear proteins. NLRP3, ASC, Caspase-1-p10, pro- and mature IL-1β, p-IκBα and IκBα, and nuclear levels of p65. Data were expressed as mean ± SEM. *n* = 5. *P < 0.05 and **P < 0.01 vs control group. ^#^*P* < 0.05 and ^##^*P* < 0.01 vs LPS group (downregulation). ^$^*P* < 0.05 and ^$$^*P* < 0.01 vs LPS group (upregulation).

To further investigate whether the antioxidative and anti-inflammatory effects of ISL on LPS-induced ALI are due to Nrf2 activation, we used WT and Nrf2^-/-^ C57BL/6 mice, and the western blot results confirmed the knockout of Nrf2 (Figure 9A). Histological analysis provided a clear impression of the protective effect: ISL notably alleviated the histopathological changes in WT mice, but the inhibitory effect was fairly weaker in the Nrf2^−/−^ mice (Figure 9B). Furthermore, ISL significantly suppressed the LPS-induced ROS production in peritoneal macrophages of WT mice but not in the peritoneal macrophages of the Nrf2^−/−^ mice (Figure S3 in Supplementary Material), showing that the antioxidative capacity of ISL was dependent on Nrf2 activation. In addition, in accordance with the *in vitro* experiments, the inhibitory effect of ISL on the NLRP3 pathway was substantially blocked in Nrf2^−/−^ mice compared with WT mice, while the inhibitory effect on the NF-κB pathway still existed (Figure 10). Meanwhile, in the Nrf2^−/−^ mice, we were surprised to find that the ISL + LPS group had increased expression levels of the NLRP3 inflammasome components compared with their levels in the LPS group, but the reason remains unclear. Such findings further support the results that ISL alleviates LPS-induced ALI through the inhibition of NLRP3 in a Nrf2-dependent way and the suppression of the NF-κB pathway in a Nrf2-independent way.

## Discussion

**Figure 11:**
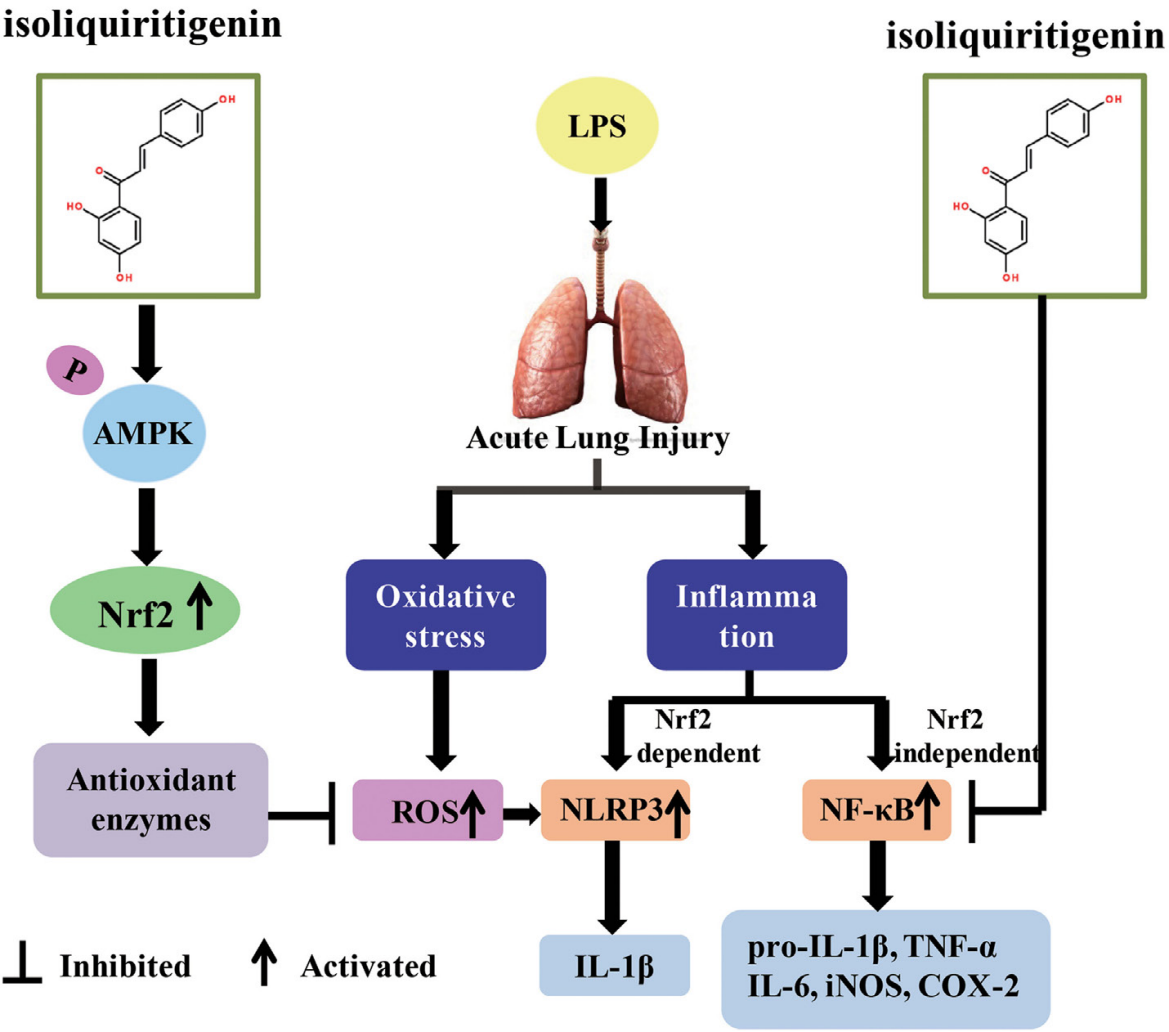
Scheme summarizing the protective effect of isoliquiritigenin (ISL) on lipopolysaccharide (LPS)-induced acute lung injury (ALI) and the underlying mechanisms. ISL treatment significantly alleviated LPS-induced ALI via the restriction of oxidative and inflammatory injury, which was associated with activation of AMP-activated protein kinase (AMPK)/Nrf2/antioxidant response element signaling and inhibition of NOD-like receptor protein 3 (NLRP3) and NF-κB pathways. Nevertheless, the inhibition of NLRP3 and NF-κB pathways by ISL could be Nrf2-dependent or Nrf2-independent, respectively.

Oxidative stress represents a perturbed redox equilibrium and is reported to have an interdependent relationship with inflammation (2, 7). It is intimately involved in multiple diseases, including ALI/ARDS, a major clinical syndrome characterized by diffuse inflammation and respiratory failure (23, 28). For protection against oxidative stress and relevant inflammation, organisms have developed a complex cellular defense system and Nrf2 is an important component of that system. ISL, a flavonoid from *G. uralensis*, possesses diverse biological properties, including antioxidant and anti-inflammatory activities, which may be due to its regulation of Nrf2 and other pro-inflammatory pathways (26, 27). The present study revealed the protective effect of ISL on ALI/ARDS and the underlying mechanisms of this effect, including the regulation and crosstalk of the Nrf2, NLRP3, and NF-κB pathways (Figure 11).

To imitate the environment of oxidative stress in ALI, t-BHP is widely used to induce ROS *in vitro*, and LPS-related models are usually used *in vivo* (28, 29). Excessive ROS mediates epithelial and endothelial injury in the lung (40, 41) and promotes tissue destruction, such as histopathological changes, lung edema, and protein leakage in ALI. It is widely accepted that the transcription factor Nrf2 effectively reduces ROS levels and combats redox disruption through the induction of ARE-dependent genes (8). Moreover, Nrf2 is considered a candidate susceptibility gene in several ALI model and has notable benefits in ALI/ARDS (42). In the current study, ISL also increased the expression levels of nuclear Nrf2 and its target antioxidant enzymes including HO-1, NQO1, GCLM, and GCLC. At the same time, excessive ROS was indeed eliminated by ISL and was accompanied by reductions in t-BHP-stimulated cell death *in vitro* and in LPS-induced MPO/MDA production, GSH/SOD depletion and lung injury *in vivo*. Therefore, the alleviation of cytotoxicity and lung injury by ISL might be linked to its ability to counterbalance excessive ROS through Nrf2 signaling.

Furthermore, AMPK, an energy sensor that regulates cell survival or death, can also be activated by ISL and exert cytoprotective effects (10, 43). Recently, crosstalk between Nrf2 and AMPK has been reported in human endothelial cells, and AMPK is considered to work as an upstream kinase of Nrf2 (44, 45). Moreover, certain compounds activate AMPK by increasing its phosphorylation level, subsequently promoting the Akt-mediated phosphorylation of GSK3β and eventually leading to Nrf2 activation (11). Although previous studies have also shown that CC may exert effects independent of AMPK inhibition, such as inhibit the BMP pathway, UPR transcription program, and antiglioma, it is still the only available cell-permeable AMPK inhibitor (46–49). Using CC, the present study results suggested that ISL activated Nrf2 in an AMPK-dependent way in macrophages. Meanwhile, the increased phosphorylation of AMPK, GSK3β, and Akt was also observed in ISL-treated mice. The ability of AMPK to regulate Nrf2 further indicated that energy metabolism dysfunction might interact with oxidative stress and serve as a danger signal in ALI, while the influence of ISL on energy metabolism and oxidative stress may satisfy the prerequisites of each other.

Nevertheless, persistent and uncontrolled inflammation is also intimately associated with oxidative stress and implicated in LPS-induced ALI (50). LPS promotes the infiltration of inflammatory cells and pro-inflammatory mediators to evoke more production of ROS; in turn, excessive ROS inflicts more severe inflammation, exacerbating tissue destruction (51, 52). As reported, the severity of ALI is correlated with two interdependent processes: the recruitment of inflammatory cells and the upregulation of pro-inflammatory cytokines (53–55). ISL significantly inhibited LPS-induced inflammation, including the infiltration of inflammatory cells (neutrophils, macrophages) and the overproduction of two important pro-inflammatory enzymes, COX-2, and iNOS, and other pro-inflammatory cytokines (TNF-α, IL-1β, and IL-6). Clearly, in addition to its antioxidative property, the ISL-mediated alleviation of lung injury was also derived from the reduction of these inflammatory components.

Among the various inflammatory responses, two pathways are particularly required for lung injury: the NF-κB and NLRP3 pathways. NF-κB, a transcription factor mainly composed of the p50/p65 heterodimer, plays a crucial role in regulating pro-inflammatory cytokines. Under unstimulated conditions, NF-κB interacts with its inhibitor protein IκB and is sequestered in the cytosol. Upon stimulation, IκB is phosphorylated and degraded, and then, NF-κB is released, phosphorylated and translocated into the nucleus to trigger cytokine precursors (56, 57). Furthermore, the activation of NF-κB is an essential initial step for the priming of NLRP3 activation, and ROS generated from NF-κB-mediated inflammation also serves as a danger signal that activates NLRP3 (18, 21). Once NLRP3 is activated, there ensues the recruitment of ASC, activation of caspase-1, and processing of pro-IL-1β or pro-IL-18 into mature forms ensues (19). Thus, these two separate signals act together to induce the activation of cytokines such as IL-1β, promoting lung injury in ALI. The present study verified the inhibitory effects of ISL on both the NF-κB and NLRP3 pathways. This dual inhibitory effect might act at different steps in the pathways alleviating lung injury in LPS-induced ALI, and was more effective than the inhibition of a single pro-inflammatory signaling pathway.

Moreover, recent studies have also revealed that Nrf2 activation is able to repress inflammation-mediated injury through its antioxidant cascades (38). However, the relationship between Nrf2 and pro-inflammatory pathways, such as the NF-κB and NLRP3 pathways, is still controversial in several studies (58–61). In the present study, we also investigated the crosstalk between Nrf2 and these two pathways. Brusatol (a specific inhibitor of Nrf2) pretreatment abolished the protective effects of ISL on cell viability and ROS in RAW 264.7 cells, and the inhibition of ROS was also abolished in the peritoneal macrophages of Nrf2^−/−^ mice. The inhibitory effect of ISL on NLRP3 activation was markedly reduced when cells were treated with brusatol and in the lung of Nrf2^−/−^ mice, while the inhibition of IκBα degradation was not affected. This suggests that ISL might attenuate LPS-induced ALI by inhibiting the NLRP3 pathway in a Nrf2-dependent way and the NF-κB pathway in a Nrf2-independent way. Hence, the impact of ISL on these two pro-inflammatory pathways may act, respectively, and NF-κB inhibition might not be adequate to dampen LPS-induced lung injury. More intriguingly, ISL treatment significantly alleviated the histopathological changes in WT mice, while this protective effect was fairly weaker in the Nrf2^−/−^ mice. On one hand, these results indicate that the anti-inflammatory activity of ISL was at least partly dependent on Nrf2; on the other hand, the NLRP3 pathway might be regulated by other priming signals apart from NF-κB and play a much more important role in the full development of ALI.

In conclusion, we first demonstrated that ISL significantly alleviated LPS-induced ALI in mice and that the mechanisms underlying this protective effect might include the restriction of oxidative damage and inflammatory injury, which was derived from the activation of AMPK/Nrf2/ARE signaling and the inhibition of the NF-κB and NLRP3 pathways. Additionally, although the antioxidative effect of ISL is mainly derived from Nrf2 activation, the inhibitory effect of ISL on LPS-induced inflammation may be either Nrf2-dependent or Nrf2-independent. In summary, the present results provide experimental evidence for the application of ISL in the treatment of ALI/ARDS associated with gram-negative bacterial infection.

## Ethics Statement

All animal experiments were performed according to the guide for the Care and Use of Laboratory Animals, which was published by the US National Institute of Health.

## Author Contributions

QL conducted the experiments and wrote the paper; HL conducted the experiments; ZW analyzed the data, XC and LP contributed to the design and improvement of the experiments.

## Conflict of Interest Statement

The authors declare that the research was conducted in the absence of any commercial or financial relationships that could be construed as a potential conflict of interest.

## Supplementary Material

Figure S1 Effects of isoliquiritigenin on t-BHP-induced oxidative injury in RAW 264.7 cells. **(A)** RAW 264.7 cells were treated with isoliquiritigenin (5, 10, 20 µM) for 18 h with/without BT (300 nM), and then subjected to t-BHP (10 mM) for 3 h. The cell viability was determined by a MTT assay. **(B)** RAW 264.7 cells were treated with isoliquiritigenin (5, 10, 20 µM) for 18 h with/without BT and then stained with DCFH-DA (5 µM) for 40 min, followed by exposed to t-BHP (10 mM) for 5 min to induce reactive oxygen species (ROS). The ROS production was detected by fluorescence microplate reader. Data were expressed as mean ± SEM. *n* = 5. **P* < 0.05 and ***P* < 0.01 vs control group. ^#^*P* < 0.05 and ^##^*P* < 0.01 vs t-BHP group.

Figure S2 Inhibition of Nrf2-target antioxidative enzymes by Brusatol. RAW 264.7 cells were treated with BT (Brusatol, an inhibitor of Nrf2, 300 nM) for 1 h and then subjected to isoliquiritigenin (20 µM) for 6 h. The inhibition of HO-1, GCLM, GCLC, and NQO1 expression were determined by western blot and expressed as densitometry quantitation using β-actin as an internal control. Data were expressed as mean ± SEM. *n* = 5. **P* < 0.05 and ***P* < 0.01 vs control group. ^#^*P* < 0.05 and ^##^*P* < 0.01 vs isoliquiritigenin only group.

Figure S3 Nrf2 dependence of antioxidative effects mediated by isoliquiritigenin in peritoneal macrophages. Peritoneal macrophages isolated from wild-type (WT) and Nrf2^−/−^ mice were pre-treated with isoliquiritigenin (20 µM) for 1 h and then stimulated with lipopolysaccharide (LPS) for another 24 h. After that, cells were stained with DCFH-DA (5 µM) for 40 min, and reactive oxygen species production was detected by fluorescence microplate reader. Data were expressed as mean ± SEM. *n* = 5. **P* < 0.05 and ***P* < 0.01 vs control group. ^#^*P* < 0.05 and ^##^*P* < 0.01 vs LPS group.
