# Supplementary figures and images for: Isoliquiritigenin Activates Nuclear Factor Erythroid-2 Related Factor 2 to Suppress the NOD-Like Receptor Protein 3 Inflammasome and Inhibits the NF-κB Pathway in Macrophages and in Acute Lung Injury
Source: Front Immunol. 2017 Nov 9;8:1518. doi: 10.3389/fimmu.2017.01518 (PMC5677786; doi:10.3389/fimmu.2017.01518)

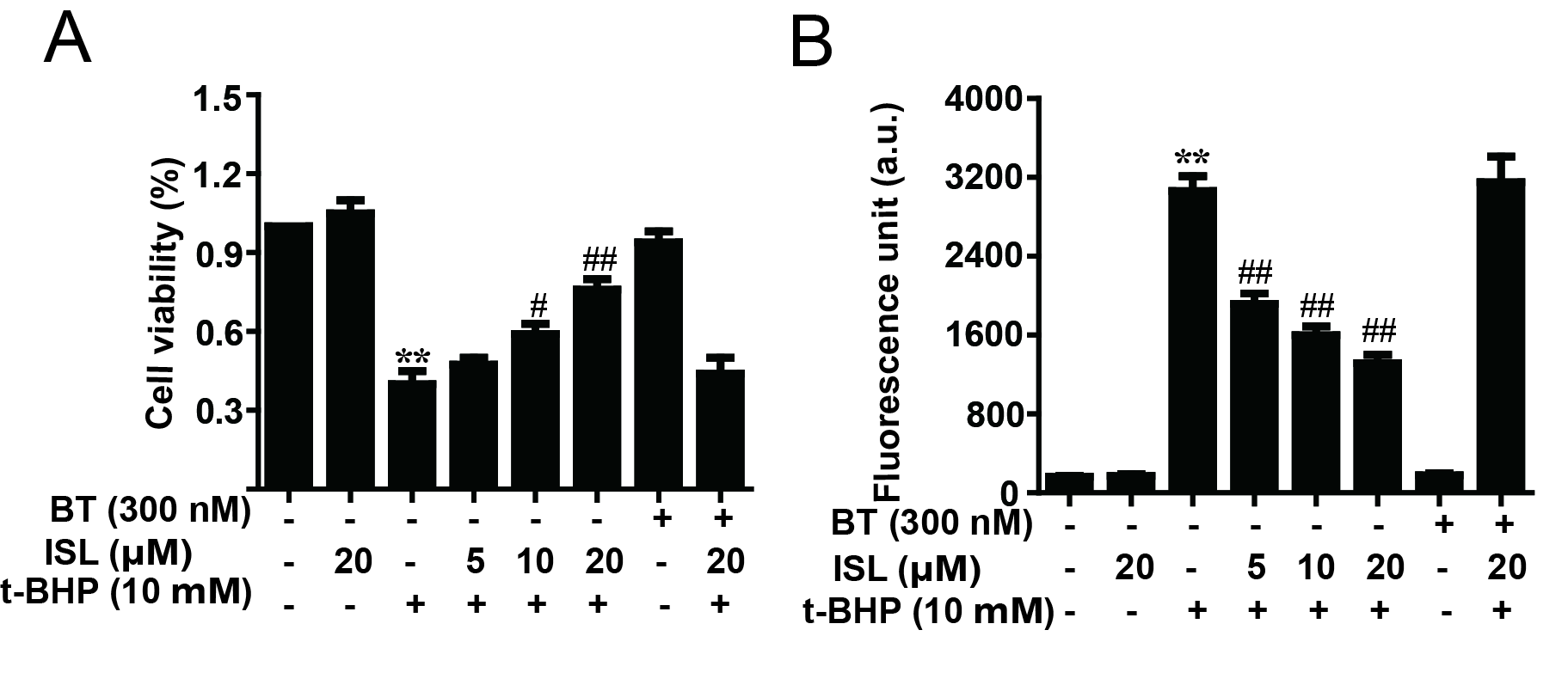

Supplement: Figure S1 — Effects of isoliquiritigenin on t-BHP-induced oxidative injury in RAW 264.7 cells. (A) RAW 264.7 cells were treated with isoliquiritigenin (5, 10, 20 µM) for 18 h with/without BT (300 nM), and then subjected to t-BHP (10 mM) for 3 h. The cell viability was determined by a MTT assay. (B) RAW 264.7 cells were treated with isoliquiritigenin (5, 10, 20 µM) for 18 h with/without BT and then stained with DCFH-DA (5 µM) for 40 min, followed by exposed to t-BHP (10 mM) for 5 min to induce reactive oxygen species (ROS). The ROS production was detected by fluorescence microplate reader. Data were expressed as mean ± SEM. n = 5. *P < 0.05 and **P < 0.01 vs control group. #P < 0.05 and ##P < 0.01 vs t-BHP group. [file image_1.tif]

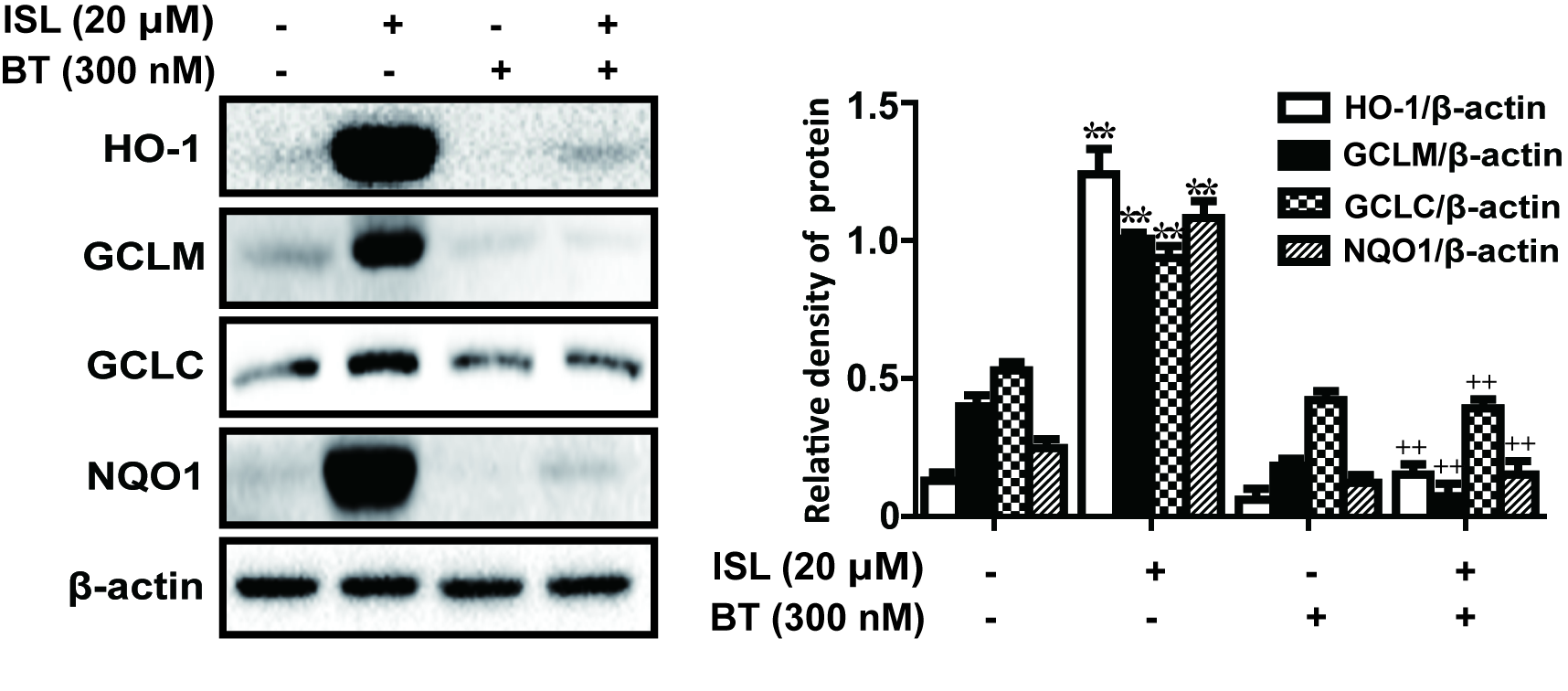

Supplement: Figure S2 — Inhibition of Nrf2-target antioxidative enzymes by Brusatol. RAW 264.7 cells were treated with BT (Brusatol, an inhibitor of Nrf2, 300 nM) for 1 h and then subjected to isoliquiritigenin (20 µM) for 6 h. The inhibition of HO-1, GCLM, GCLC, and NQO1 expression were determined by western blot and expressed as densitometry quantitation using β-actin as an internal control. Data were expressed as mean ± SEM. n = 5. *P < 0.05 and **P < 0.01 vs control group. #P < 0.05 and ##P < 0.01 vs isoliquiritigenin only group. [file image_2.tif]

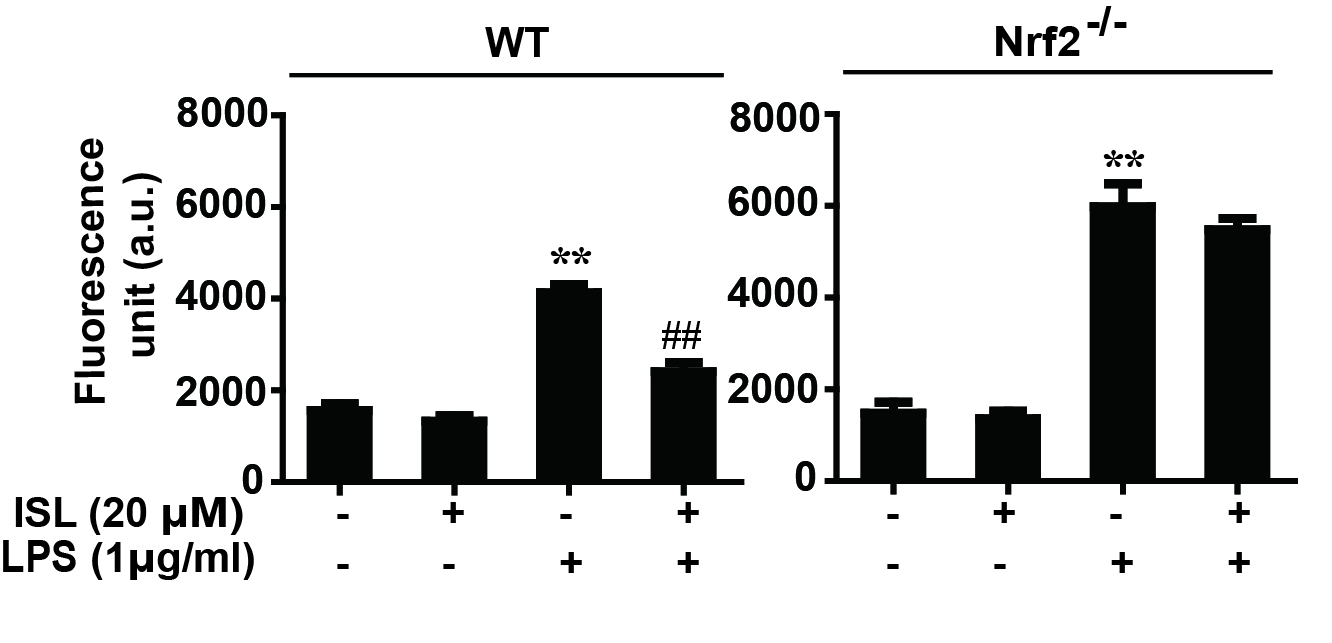

Supplement: Figure S3 — Nrf2 dependence of antioxidative effects mediated by isoliquiritigenin in peritoneal macrophages. Peritoneal macrophages isolated from wild-type (WT) and Nrf2−/− mice were pre-treated with isoliquiritigenin (20 µM) for 1 h and then stimulated with lipopolysaccharide (LPS) for another 24 h. After that, cells were stained with DCFH-DA (5 µM) for 40 min, and reactive oxygen species production was detected by fluorescence microplate reader. Data were expressed as mean ± SEM. n = 5. *P < 0.05 and **P < 0.01 vs control group. #P < 0.05 and ##P < 0.01 vs LPS group. [file image_3.tif]
